# Patient and Public Involvement and Engagement within a UK blood cancer cohort: a case study

**DOI:** 10.1186/s40900-025-00829-6

**Published:** 2026-01-20

**Authors:** Debra Howell, Carol Miller, Amy Rebane, Rebecca Sheridan, Eve Roman, Alexandra Smith

**Affiliations:** 1 Epidemiology & Cancer Statistics Group, Department of Health Sciences, University of York, York, YO10 5DD UK; 2 Member of the Public, Private Address, UK; 3 Leeds NIHR Biomedical Research Centre, Chapel Allerton Hospital, Leeds, LS7 4SA UK

**Keywords:** Involvement, Engagement, Patient, Public, Carers, Research, Healthcare, Blood cancer

## Abstract

This paper presents a case study of Patient and Public Involvement (PPI), in which people shared their ‘lived experience’ as ‘experts’ who manage their blood cancer on a daily basis, within their own environment and social circumstances. The case study is set within an ongoing cohort study that was established in 2004 in the North of England, and currently includes ~ 55,000 newly diagnosed patients (no exclusions), increasing annually by ~ 2,500. Participants are invited to join a Patient Partnership, and if they agree may be asked to take part in PPI. PPI is conducted with selected patients, matched to the research question. It takes place during one-to-one or group consultations (interviews or focus groups), which may occur face-to-face or online, and during the daytime or evening, on a weekday or weekend, to provide various options and promote inclusivity. PPI meetings have generated advice on research priorities, website design and content, and study design and implementation; with more formal collaborations established within funding applications, and Oversight Committee and Sub-Committees. Researchers received practical assistance in preparing clear study paperwork written in plain language, for information leaflets and consent forms, academic papers, and summaries used to share findings on lay-websites and social media sites. Letters of support have also been provided for funding applications. PPI has underpinned the cohort’s ethical and governance approvals; and improved its methods, and the way in which findings are shared. Open Days and other meetings with patients and the public have raised awareness about the cohort and engaged people in the research process. People said they were pleased to take part in PPI and to have the opportunity to give something back. Factors for success were the inclusion of appropriate stakeholders, sufficient reimbursement, feedback on impact, skilled facilitators, and good governance. PPI activities will be strengthened within the cohort over coming years, by significant expansion in members of the Oversight Committee and Sub-Committees, from across the study area, and with an increased focus on underserved communities. Effective PPI requires sufficient time, skills, effort and resources; and it is important to be aware of changes in this rapidly developing area.

**Supplementary Information:**

The online version contains supplementary material available at 10.1186/s40900-025-00829-6.

## Abstract (plain language summary)

Patient and Public Involvement (PPI) is when people (e.g., patients, potential patients, caregivers) work with researchers to improve clinical knowledge and NHS healthcare. This paper presents a case study showing how PPI was set up to improve the lives of people affected by blood cancer in an ongoing study, started in the North of England in 2004. The study now includes 55,000 blood cancer patients, all of who have data collected from their medical records. They are also invited to join a Patient Partnership, and those who agree may be asked to take part in PPI. PPI has involved people affected by blood cancer sharing their views as ‘experts’ with ‘lived experience’. These voices have helped decide what studies to carry out and how best to do them. This makes sure the cohort’s work is important, easy to take part in and able to improve NHS care. PPI is carried out in one-to-one or group meetings that may be face-to-face or online, to give people have different options. Patients and the public help researchers prepare study paperwork and websites; join funding applications and committees, and give advice. People affected by blood cancer have worked with the research team to write up findings in plain words, for sharing with the public on website and social media sites; and they have taken part in our Open Days and other meetings. This raises awareness about the study and helps people get involved in research. Time, skills and funding are needed for good PPI.

**Supplementary Information:**

The online version contains supplementary material available at 10.1186/s40900-025-00829-6.

## Background

Patients and Public Involvement (PPI) in healthcare research is advocated by policymakers, researchers and patients, and mandatory for some funders and scientific journals [1, 2]. PPI’s concept and terminology is ever-evolving, with little consensus about precise meanings and methods; and limited reporting, that is inconsistent in quality [3–5]. The UK’s National Institute for Health and Care Research (NIHR) discusses ‘Public Involvement’, which it defines as research conducted ‘with’ or ‘by’ the public, rather than ‘to’, ‘about’ or ‘for’ them; with the ‘Public’ including patients, potential patients, carers and people who use health services [6, 7]. In this approach, public collaborators work alongside researchers to determine: ‘what research gets done’, ‘how it is carried out’ and ‘how the results are shared and applied in practice’ [6–10]. It contrasts with ‘Participation’, in which patients take part in research, but do not input into study design or management [7, 9, 10]. This paper refers to PPI, while encompassing NIHR definitions [6, 7].

Now common practice in the UK, PPI is considered ‘important, expected and possible’ [11, 12], with benefits including shared knowledge and insights from real-world ‘lived experience’ perspectives provided by ‘experts by experience’ [2, 9, 13]. Its purpose is to improve the efficiency, acceptability and value of research by increasing its relevance to patients; supporting recruitment and retention; and promoting equality, diversity, inclusion and dissemination beyond academia [2]. It is concerned with increasing accountability and transparency, and the democratisation of health and medical research, whether it be funded by public money [2, 9], or by charitable organisations. It may also be seen as a *‘*right’ and ‘duty’ by the public, enabling the voices of underserved groups to be heard, power to be balanced with researchers, and knowledge to be co-constructed [2, 11].

Different types of PPI are recognised, ranging from that which is consultative (seeking views on aspects such as the research question), collaborative (ongoing partnership across the research process) or ‘publicly led’ (where the public designs and undertakes the study) [5]. ‘Co-design’ and ‘Co-production’ are key concepts, with ongoing partnership moving towards a sharing of responsibility and power; the logic being that those affected by the research (‘end-users’) are best placed to design and deliver it, and have equally important skills and knowledge to contribute [5, 14, 15]. National standards and benchmarks have been created to ensure effective processes, and indicators exist to monitor improvements [12]. Frameworks and reporting checklists have been developed to improve quality, transparency and consistency, and support best practice (what works, in what context, for whom, why) [2, 16].

PPI has been readily adopted by UK charities. Cancer Research UK refers to ‘Patient Involvement’ as including anyone affected by cancer, and the wider public, stating that these groups will ‘guide, influence and shape projects… to increase our understanding of cancer and ensure our work meets the needs of people affected by cancer’ [17]. Organisations targeting specific malignancies, such as Blood Cancer UK, refer to ‘Patient and Public Involvement’, which they define as involving ‘experts through lived experience of blood cancer in the planning and delivery of research projects’ [18].

With a view to informing good practice, this case study shares the methods used to instigate and develop PPI within an ongoing cohort of newly diagnosed patients with haematological malignancies (blood cancers, including various subtypes of leukaemia and lymphoma, and also myeloma). The aims and infrastructure of the cohort itself are first described; followed by the PPI case study, which details the process of establishing a core group from which to source people for PPI, the activities undertaken, the impact and benefit from these, and the challenges and successes. This case study is intended to inform other researchers who may just be starting out on their PPI journey.

### The cohort study

With different symptoms, treatments, and outcomes, blood cancers are the fifth most frequently diagnosed cancer worldwide [19, 20]. Affecting both sexes and all ages, the diverse characteristics of this group afford a particularly useful exemplar from which to consider PPI, while providing information that could be relevant to other cancers and conditions.

**Fig. 1:**
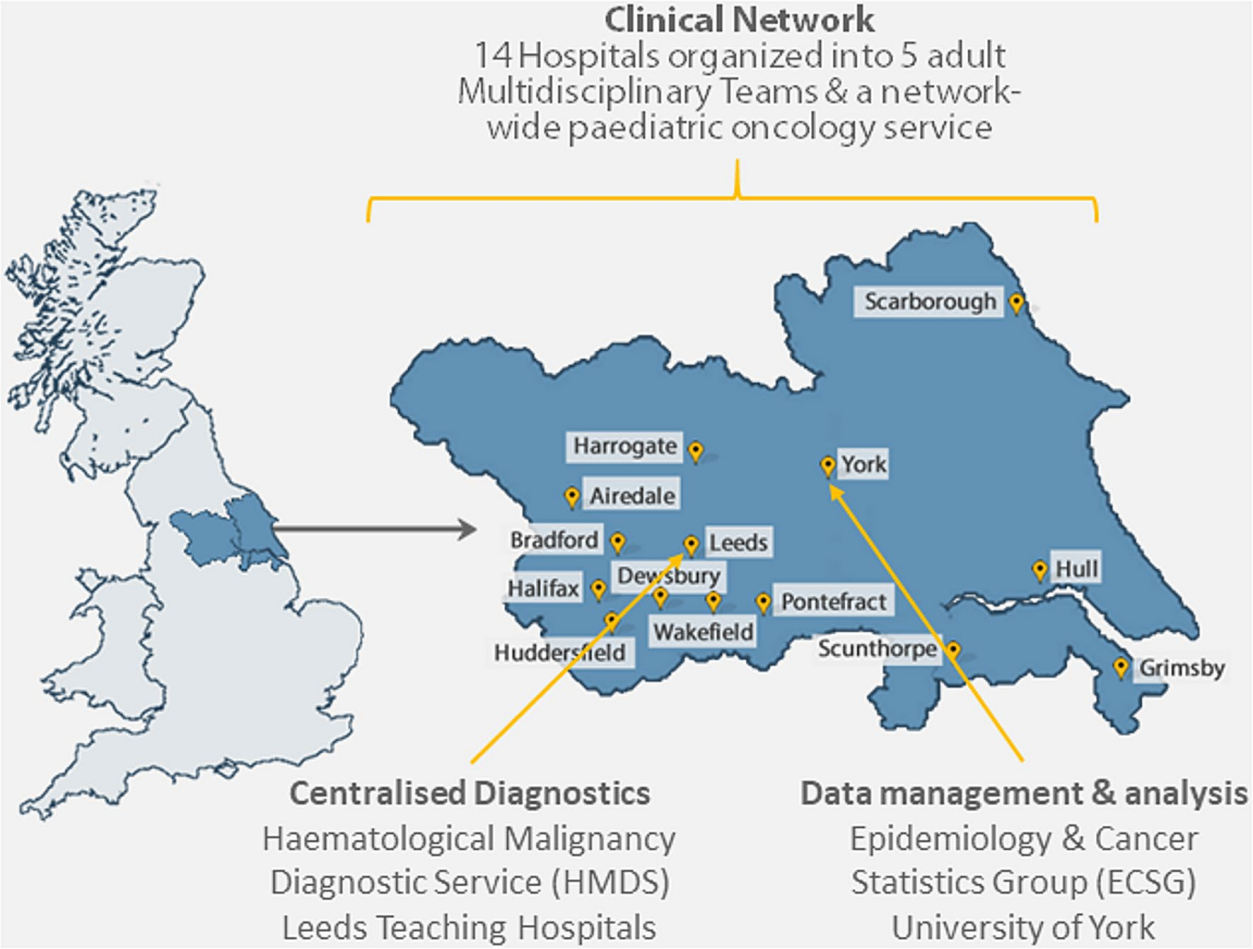
Cohort setting and clinical care

The cohort (known as the Haematological Malignancy Research Network (HMRN): www.hmrn.org) was established in 2004 in the North of England, and covers a population of ~ 4 million people (Fig. 1). Briefly, it aims to improve the knowledge, NHS care and lives of people affected by blood cancer. It is an ongoing project and currently holds data on around 55,000 patients; increasing by ~ 2500 annually, with no exclusions by protected characteristics [21, 22]. HMRN has a comparable distribution by sex and age, urban/rural status and area-based deprivation (Index of Multiple Deprivation, income domain) to that of the UK as a whole [23–25].

Within the catchment, all blood cancers are diagnosed by the Haematological Malignancy Diagnostic Service (www.hmds.org); and care is provided by 14 hospitals (Fig. 1), in adherence with national guidelines. All patients are tracked from diagnosis and have information collected from their NHS records, with data quality maintained via rigorous standard operating procedures (see **Sect. 3.1.3** for further information). The data are used in epidemiological research and for clinical audits [24, 25]. Additional qualitative research is conducted from within the cohort on the lived-experiences of people affected by blood cancer [26–28]. Researchers at the University of York oversee all data governance, and all data collection and analyses.

HMRN’s ethical approval has been in place since 2004, viaLeeds West Ethics Committee (04/Q1205/69). Section 251 support from the ConfidentialityAdvisory Group (CAG; NHS Act 2006: 20/CAG/0149) provides the legal basis for datacollection, and each person is assigned a unique ID and linked to national data on death,cancer and Hospital Episode Statistics.

## Patient and Public Involvement (PPI)

From instigation of the cohort, PPI was considered crucial to HMRN’s success, and the impact of the research conducted within it. It began small, and has expanded and developed over the twenty years or so since the cohort began; hence it is now well-established and robust. It was intended to thread through all aspects of the cohort’s research and collaborations, and has succeeded in this respect.

### Practical preparations

**Fig. 2:**
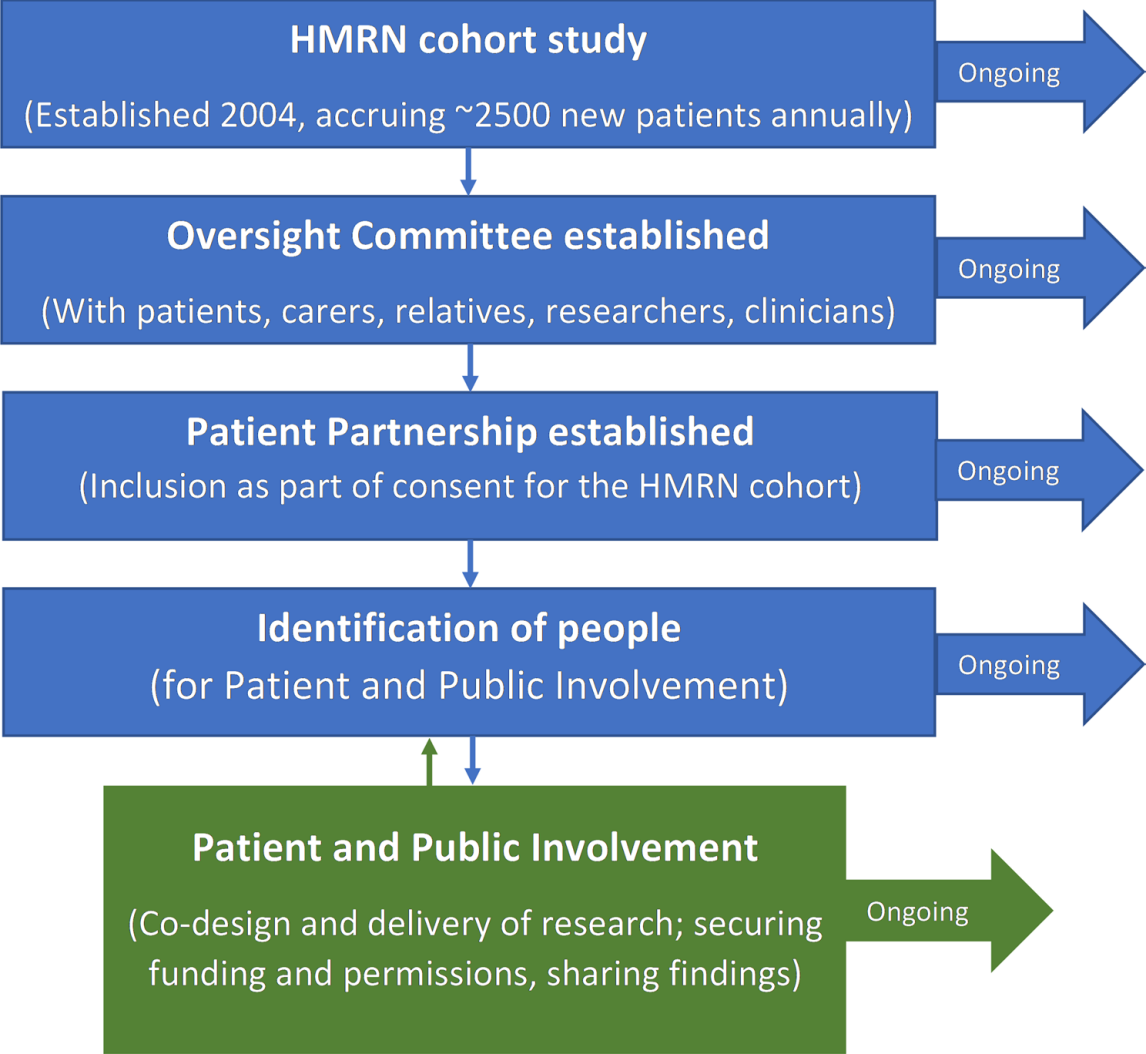
Practical preparations for Patient and Public Involvement

A number of preparations were required before individuals could be recruited for PPI, to put the necessary infrastructure into place, as described in the following section and depicted in Fig. 2.

#### Setting up an oversight committee

The first step was to locate individuals to work with the research team to establish and monitor PPI. As a result, the team decided to create a HMRN PPI Oversight Committee (Fig. 2) and reached out to the Haematology Clinical Involvement Leads in the study area for this purpose. Patients and the public were initially identified by the clinical staff, and the assembled Committee also comprised of researchers and NHS staff. Its remit was to enable lay-voices to contribute to HMRN, for example by providing advice on ethical and governance issues (e.g., data collection without consent, patient complaints) instigating its own research (e.g., on information needs), and monitoring and advising on all patient-related work (e.g., optimal recruitment methods, completion of planned research, dissemination of findings) - see Sect. “Impact and Benefits of Patient and Public Involvement”. The Committee went on to meet several times a year, but chose not to have formal Terms of Reference, although meetings were minuted and ratified. People remained on the Committee for as long as they felt they were able, and new lay-members were recruited over time, as described in “The Patient Partnership”.

#### The patient partnership

Thought was next given to the best way to find a group of patients that we could use to source people for PPI over the long-term for future research, within respectful, equal relationships. A decision was made to do this as part of the general consenting process for the HMRN cohort itself, by simply including a question on the consent form asking if the individual could be contacted again by a member of the study team. Those who agreed became part of the *Patient Partnership* (Fig. 2), which has gone on to underpin all of the cohort’s future contact with patients and family members.

#### Sourcing people for PPI

**Fig. 3:**
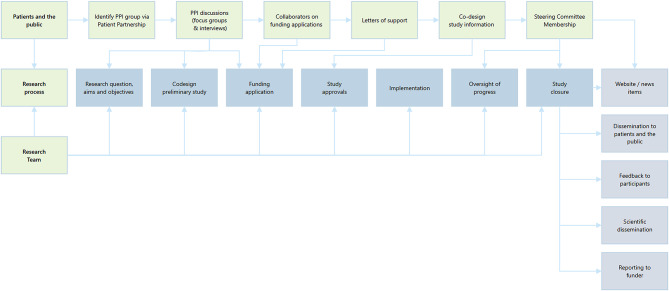
Patient, public and researcher activities along the research process

The Patient Partnership now contains thousands of people affected by blood cancer, who can be invited to join the HMRN Oversight Committee; or to collaborate with the research team on the codesign and conduct of specific projects (see Fig. 2, with an overview of activities provided in more detail in Fig. 3). Importantly, HMRN has no exclusions, meaning all newly diagnosed patients are part of the cohort, regardless of their protected characteristics [21, 22]. Furthermore, routine data collection means that each patients’ demographic (age, sex, ethnicity and socio-economic status), diagnostic (subtype, time since diagnosis), and prognostic status was known; along with all treatment(s) and outcome(s) from diagnosis onwards. As a result, patients from the Partnership could be sourced for PPI according to the characteristics that align with the research question (e.g., by diagnostic subtype, treatment type, age range). In this way, the people targeted for PPI are better able to contribute, as the research team is aware that they have had relevant lived experience. The Partnership has also been also used to recruit patients as research participants (Sect. “Background”); rather than to steer projects.

The exact number of people in the Partnership at any one time is dynamic; changing with the inclusion of newly diagnosed patients, and the loss of those with poorer outcomes. The number of individuals selected for PPI varies according to requirements, with recruitment taking place as and when necessary. The Oversight Committee has previously included up to six patients and caregivers, but is about to expand markedly, to around 20 individuals, recruited across different disease and age groups, and at various points in their treatment pathway. The number of people required for specific short term projects has ranged from one to five to date. Once individuals are identified to take part in PPI, the PPI lead will ensure each member is fully informed about all aspects of the study, and the timeline for future activities and meetings, before meeting the wider group as a whole. The extent to which PPI is undertaken is not limited; although individuals are regularly asked if they want to continue, to prevent overburdening.

#### Ethical approval for PPI

HMRN has ethical approval and CAG support (see Sect. “The Cohort Study”) but this does not specifically allude to the PPI aspects of the cohort, although activities have always been reported within the paperwork submitted to both bodies. Ethical approval is not considered mandatory for PPI in the UK [29], although researchers are beginning to question this approach and request clearer guidance [30, 31]. Given that our PPI is sourced from people affected by blood cancer, who may be feeling emotional and vulnerable (see Sect. “Factors for Success and Challenges”), this is something we intend to describe more fully in future ethics applications; for both the cohort and studies based within its infrastructure (e.g., the qualitative portfolio of work).

#### Reimbursement

HMRN’s PPI has always been fully costed within funding applications, to accurately reflect the time and effort people commit to this role. Reimbursement is typically provided in accordance with the UK’s NIHR payment guidance [32] (or guidance from other funders with their own recommendations). The amount requested is determined from the type of input required, which has become much more clearly defined in recent years. In the past, reimbursement has been requested for example, for meeting preparation and attendance; and for commenting on study material and funding applications. Resources such as childcare, transport costs, equipment and training have also been costed, to promote inclusivity and ensure everyone who wants to be involved, can be. We have recently provided computer equipment for patients and the public, and steered them towards online PPI training courses, which are often free of charge. More recently, we were asked by the NIHR to increase the PPI budget in a funding application, indicating the importance with which appropriate reimbursement is viewed.

We found that people do not always want to be compensated for their time and expenses, however, with one patient saying he asked the consultant “Is there anything I can do? I would really like to do something, to give something back [in return for NHS treatment]”. This patient wanted “to tell [others] of my experience… they should know um, what happens and what can happen… from a patient’s perspective”. As a result, this patient joined one of HMRN’s PPI groups. Reimbursement is now typically offered as vouchers, enabling individuals a choice of whether to take or refuse this. Guidance has been found to change over time, and to vary across institutions and charities, with some now insisting on BACS payment where possible, meaning the way we compensate individuals may change going forward.

##### Examples of Patient and Public Involvement across the research process

Great efforts have always been made to hear the voices of people affected by blood cancer within the HMRN cohort, and accordingly the number and range of people involved in our PPI has expanded over time. Areas in which patients and the public have been involved have also increased, with PPI contributing to all aspects of the research process, with the exception of study implementation (see Fig. 3). Findings are, however, shared with PPI members of the Oversight Committees, to ensure they resonate with patients and the public, who also prepare material with researchers for dissemination.

**Fig. 4:**
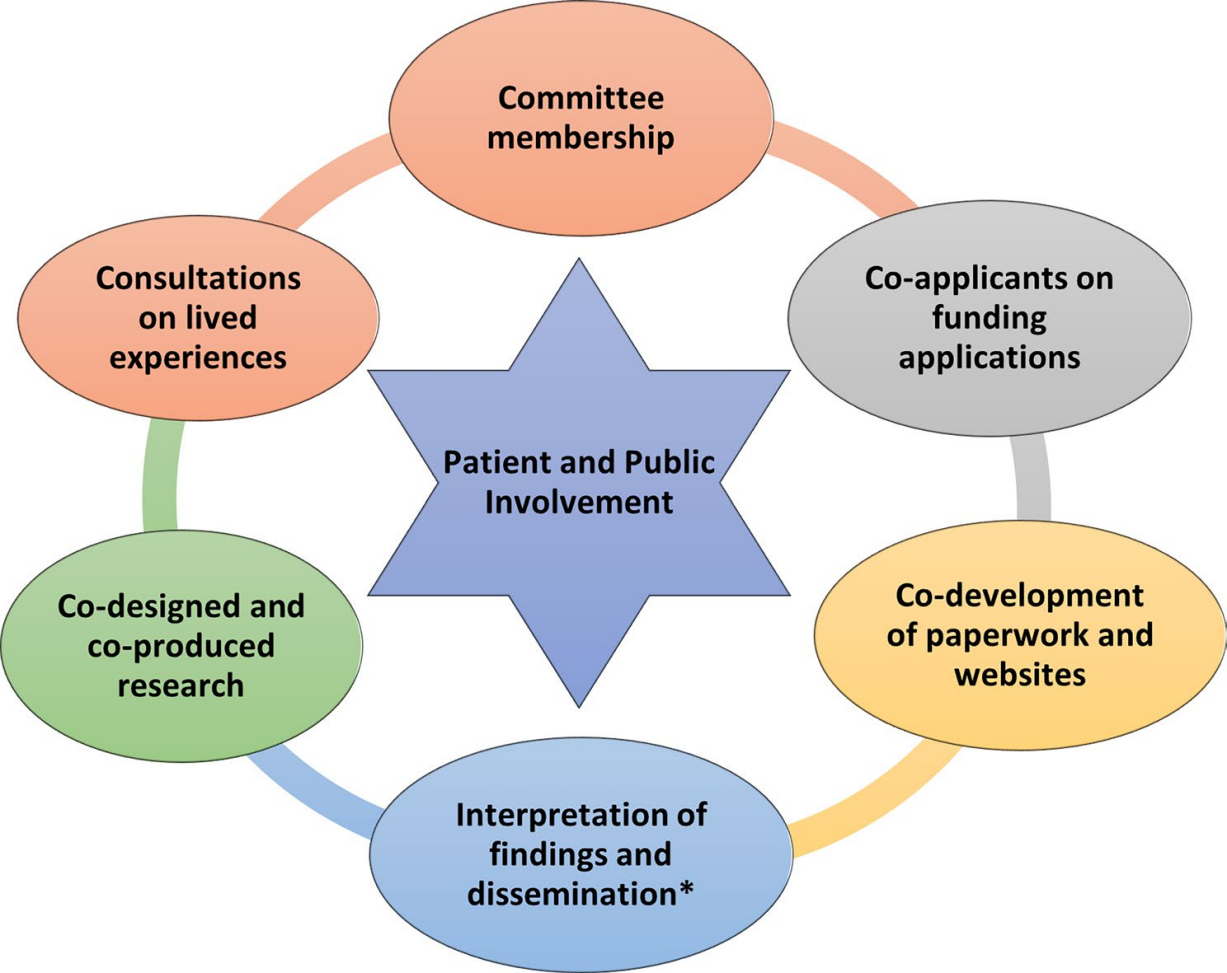
Summary of Patient and Public Involvement. *See also Fig. 5: Patient and Public Involvement to share research findings

Figure 4 summarises the ways in which people from the Patient Partnership have contributed to PPI since the cohort began in 2004. Consultations with people affected by blood cancer have been common across studies based within the HMRN infrastructure, including the qualitative research portfolio. These have typically taken the form of interviews and focus groups with members of the Partnership, who have been invited to share their lived experiences, from diagnosis to the present time. Events were always held face-to-face initially, but more recently have also been offered online, which has provided more choice and worked well, particularly for people who work during the day, or are unable to leave home or travel. Such meetings have led to the identification of priority research areas, while ensuring proposed studies are relevant, important, based on real need and potentially able to improve care. Consultations, as well as Oversight Committee meetings, have enabled researchers to discuss the cohort’s methods and ask what might be improved; an example being the option for online consenting and participation, which was recently implemented (see also Sect. “Impact and Benefits of Patient and Public Involvement” on CAG approval).

More recently patients and the public routinely co-design and deliver research in collaboration with the research team across the duration of the study, from its instigation to the dissemination of findings. This work takes place before any research plans are decided, and/or submitted for funding. Advice is requested on the topic itself and the potential research methods (such as data collection tools - e.g., food diaries, surveys, interviews; and the frequency of longitudinal input). The people involved at this stage are routinely asked to be named collaborators on funding applications, and to join the Committee associated with the study, if it is approved. Typically, the latter involves meeting two or three times annually with the research team, or more often if required, for example at the beginning and end of the project, when there is more to discuss.

**Fig. 5:**
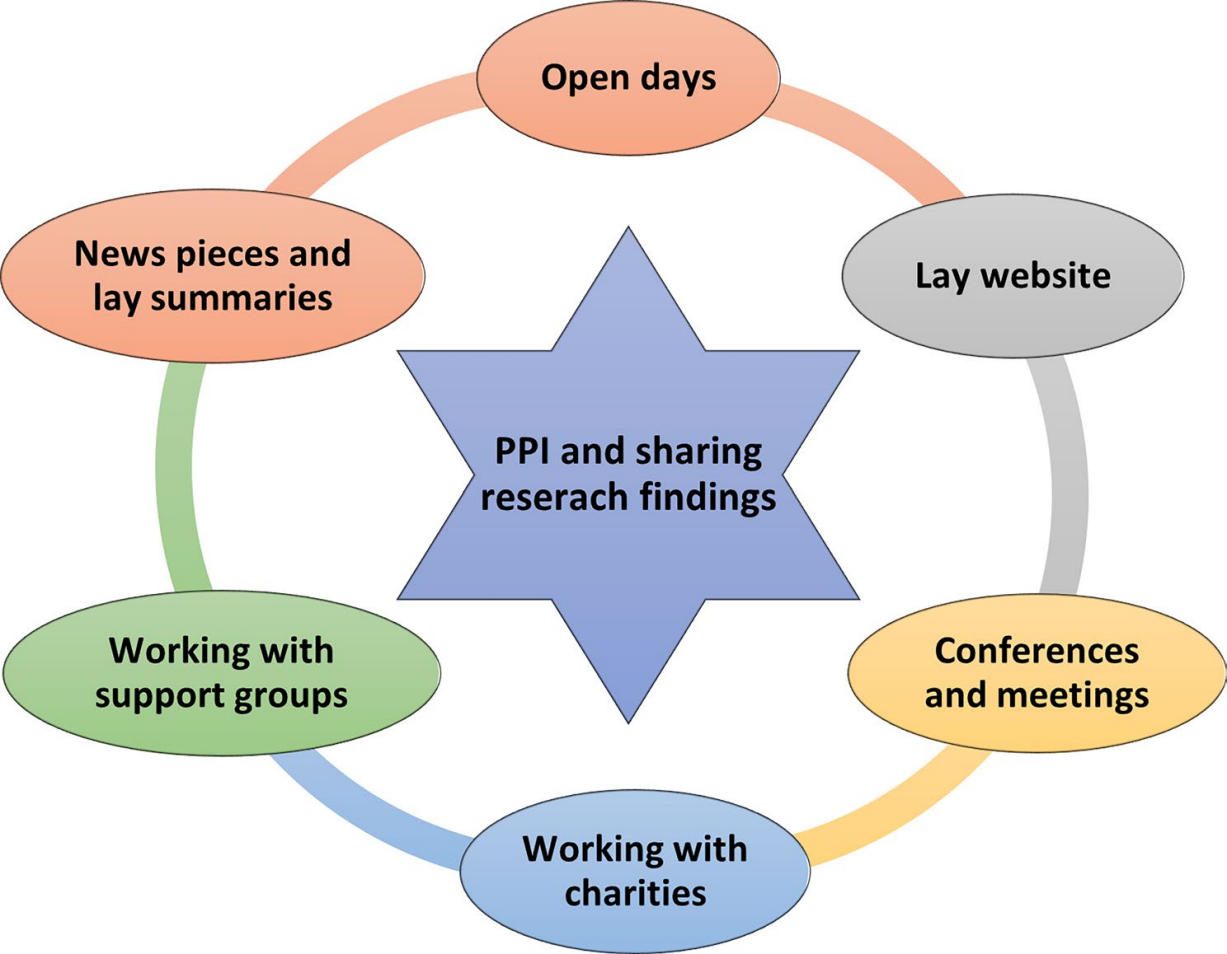
Patient and Public Involvement to share research findings

Hearing the lived experiences of people affected by blood cancer enabled researchers to demonstrate the need for further evidence about specific issues raised, and unexplained differences between blood cancers and other cancers, to ensure these were prioritised for investigation. One example is ‘Watch and Wait’; where chemotherapy only begins if/when symptoms arise, with such uncertainty often causing patients anxiety and distress. One patient described how: “it was not a nice position to be in, thinking well, there is this disease and we don’t really know how this is going to play out…”. Another said “Immediately I wanted to know, what’s the prognosis, what’s the long-term?” Some wanted personalised information about their prognosis: “(HCPs) wouldn’t talk about ‘my’ condition… they would talk about the population… that 50% figure… 50% chance of surviving 5 years is an easy thing to remember… (but) most people don’t get myeloma at 59, they get it in their mid-70s… their life expectancy is very likely less than mine”. This evidence underpinned a successful NIHR application for funding to investigate information needs and preferences within such scenarios, and how these could best be met.

A key priority area brought to the attention of the HMRN team more recently was “access to mental health services” at all stages of the cancer journey, for both patients and relatives/carers (see Sect. “Impact and Benefits of Patient and Public Involvement”). Other areas include new treatments (e.g., Immunotherapy, CAR-T therapy); the impact of treatment: “Does receiving a stem cell transplant after chemotherapy improve outcomes… what are the implications on quality of life?”; and COVID related issues (see Supplementary Material 1). Finally, PPI is currently underway (focus groups) exploring patients’ lived experiences of diet and exercise management from diagnosis through to survivorship; whilst meetings are also taking place to codesign data collection tools (e.g., food and exercise diaries) and topics to explore during in-depth interviews.

In another example, a working group was created to collaborate and consult with clinical and laboratory scientists and provide feedback about their diagnostic services, including how these might be improved to relieve anxiety for people waiting for test results. Laboratory tours were also conducted, which benefitted patients and families, who were able to learn more about blood cancer and better understand the challenges of diagnostic processes; and the scientists, who could see and talk with end-users, learn from their lived experiences and identify areas where improvements to service delivery could be made. Similarly, patients from the HMRN cohort contribute to PPI within collaborative work at associated institutions, including the NIHR Leeds Biomedical Research Centre (BRC).

A further role, as equal members of the team with lived experience of receiving information from HMRN, was the development of study material, such as information leaflets and consent forms. Iterative co-design was utilised, beginning with prototype creation and discussion, followed by ongoing discussion and modification until all stakeholders were satisfied. This input has been crucial in ensuring study material is clear and written in plain language. PPI has involved deciding the most appropriate way to share findings with patients and the public as a whole, and with the people who participated in the research itself, which is another crucial aspect of PPI. Such decisions are made as part of Oversight Committees, and have included plain language summaries that are posted or emailed to participants, or provided on the patient and public website.

In 2009, researchers were asked by a member of the Patient Partnership (CM: co-author of this paper and patient advocate) to help establish a support group for people affected by blood cancer. Although perhaps not typically considered a PPI resource, the Support Group has provided the study team with meaningful PPI at their monthly meetings, over many years. HMRN researchers have always attended these meetings (and in fact now run the group), so have been able to seek rapid PPI, which is used alongside the study-wide PPI sourced from the Patient Partnership. Input from the group has included feedback on potential studies and research findings; and the iterative co-design, development, testing and completion of an online participation portal. Individuals in the group routinely share lived experiences, provide letters of support for the cohort, and offer their time as collaborators on funding applications and as committee members. Furthermore, they provide PPI for other University based blood cancer researcher institutions (e.g., Biology and the Centre for Blood Research: on the acceptability of potential new treatments), and charities (e.g., Blood Cancer UK: on branding). The group is now well-known, with monthly meetings attended by around 30 people (patients, relatives and friends).

##### Patient and Public Involvement to share research findings

HMRN’s PPI has involved collaborating with patients and the public to share research findings, across various platforms and in different formats (see Fig. 5). One benefit from this was that people affected by blood cancer could communicate findings to their peers more appropriately and effectively than researchers; and from an empathetic stance that was derived from shared experiences. Another benefit was that patients and the public could ensure that the material shared used clear, non-clinical and non-academic language that could be understood by non-professionals.

One example was the decision to develop a HMRN website that was aimed at patients and the public, with information on the cohort’s aims, methods and study findings; which involved creating a working group from the Patient Partnership. This process utilised iterative co-design, during which initial website pages were drafted to facilitate discussion and interaction, with useful feedback provided by the group; examples including “font too small”, “better colours and navigation needed”, “feels to be aimed at academics” (see Supplementary Material 2). This was followed by ongoing interaction and modification until consensus was reached, and the Website was made live.

The website remains a key platform for sharing findings, with continued input from patients and the public, to ensure that its content remains relevant and aligned with what people want to know. The material shared includes newsletters, news items, lay-summaries of findings, and other information people said would be of interest (e.g., support groups). It also informs people how they can become involved in PPI and encourages feedback via our Freephone or dedicated study email address. We have always tried to respond to website content suggestions (e.g., ‘Patient Stories’ on topics such as “risk of infection” and what to do if “your whole-body aches” post-chemotherapy; and caregiver support, which is currently being considered). Requests could not always be met, however, especially if they were resource intensive (e.g., a “Discussion Forum” requiring ongoing moderation).

A further aspect of PPI was that of working alongside patients and the public to share HMRN’s findings outside of academia. Over HMRN’s duration, collaborations have been developed with many generic and blood cancer charities and institutions (e.g., Blood Cancer UK, Lymphoma Action, Leukaemia Care, Myeloma UK, Cancer Research UK, Marie Curie and Macmillan) (Fig. 5). These organisations have often called upon HMRN researchers to identify patients and caregivers to advocate for people affected by blood cancer, by sharing their own lived experiences at conferences and other meetings. Patients have also presented at clinical and research conferences and meetings and have also been involved in organising and running HMRN *Open Days*, presenting to the audience, and hosting stalls to discuss what it is like take part in PPI and/or provide information and support. These strategies have aimed to raise awareness about HMRN, promote engagement in its research processes and ensure people are aware of opportunities to become involved. DH also routinely visits patient Support Groups and liaises with charity representatives across the area and beyond, to discuss HMRN and its PPI groups, and to engage people in its research.

##### Impact and benefits of patient and public involvement

**Fig. 6:**
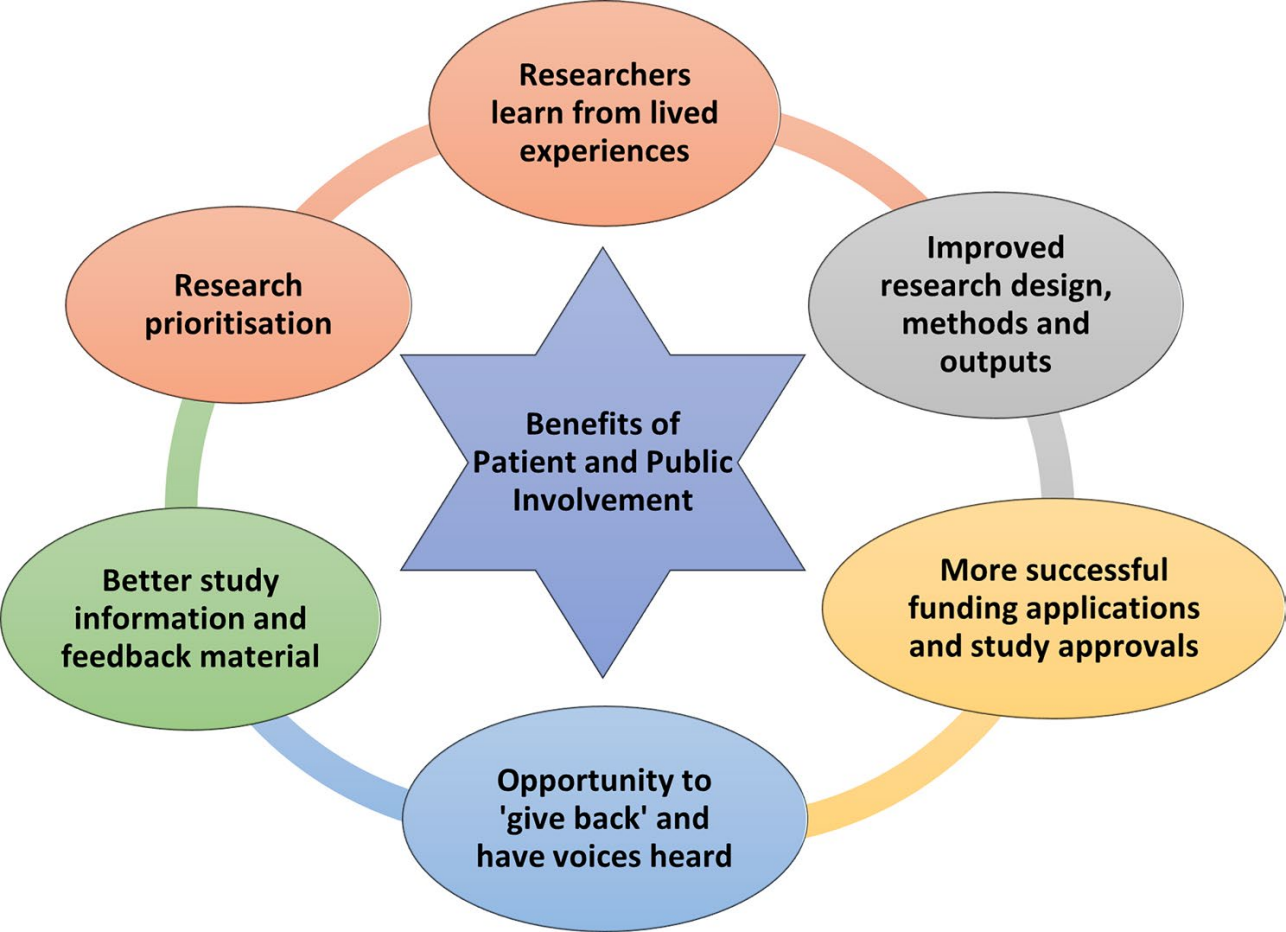
Impact and benefits of patient and public involvement

PPI impacted and benefitted patients and the public, researchers and the cohort study itself in many different ways, as is summarised in Fig. 6. Patients and relatives often told the research team that taking part in PPI had had a positive impact on them, that they had enjoyed it and, in some ways had found it beneficial. For example, they said they appreciated the chance to talk about their blood cancer experiences, and to do this in some detail and at length. Similarly, focus groups gave people the chance to informally engage with their peers (other patients and caregivers in the same situation) and discuss issues that they may not have previously shared. As noted in Sect. “Reimbursement”, they also appreciated being able to ‘give something back’ and to hear about the research that is being done on their behalf, or that could benefit others in the future. Although PPI could be emotional and trigger traumatic memories, some also described this process as cathartic.

PPI had a positive impact by enabling the research team to learn more about what it is like for individuals to live with blood cancer on a day-to-day basis, within each their own environment and social circumstances; or to care for someone with this condition. As a result, the research team were better able to direct studies towards specific areas of need and to prioritise research with more confidence (see Fig. 6). This improved all aspects of HMRN’s research, including its aims, methods, and interpretation and sharing of findings. An example is a study on CAR-T lived experiences, in which prior PPI enabled caregivers to report their own experiences and challenges, with one saying “I’m not going to, you know, make it rosy… It is very, it’s scary!”; and “you get mixed feelings mentally, and people ask… how’s it going… is he going to be alright, and all that. You have to end up saying, I don’t know. We don’t know”; and “I was ready to retire, but I would have had to, there was no way that I could have been at work. My concentration wouldn’t have been there… [as husband] was on my mind 24/7.” Such statements highlighted the significant contribution made by caregivers, and their own emotional difficulties and support needs, which resulted in their inclusion in the successful funding application as collaborators and Committee members; caregivers will also now be interviewed as study participants.

PPI has impacted positively on methods of consenting patients to HMRN. People told us they wanted to do this online, both to save resources (e.g., environmental effects) and to streamline the costs of running the study (e.g., paper, Freepost envelopes, postage charges). As noted in Sect. “Examples of Patient and Public Involvement Across the Research Process”, the online portal was co-designed and tested with patients, and is now fully operational, with around a third of patients taking part in this way, and numbers expected to increase in the future.

PPI consultations underpin all HMRN’s research and have impacted positively by providing much needed evidence about blood cancer experiences, as is clear from the well-cited publications arising on, for example, time/route to diagnosis [33–36] and symptoms/help-seeking [26, 37]. The PPI Oversight Committee (Sect. “Setting up an Oversight Committee”) previously generated impact by instigating a patient survey, and using the findings to underpin work on ‘Watch and Wait’ [27, 38], treatment [39, 40], psycho-social support [41] decision-making [42] and information needs [28, 43]. This approach encapsulated PPI across the entire research process, beginning with a co-designed questionnaire that was sent to members of the Patient Partnership, and leading to a successful funding application; and ending with a final report co-authored by a member of the PPI team [44]. More recent concerns were reported by patients and families about “the effect of COVID”, which led to the prioritisation and publication of research in this area [45]. Similarly, anxiety about ”quality of life” led to work on the health impact of premalignant disease [46]. Patients are acknowledged in all HMRN publications, and PPI is routinely described, often at the journal’s request.

PPI has been an integral part of the permissions HMRN needed to implement the cohort study. It has improved the information sent to patients, increasing the likelihood that the study would be granted ethical approval. It also enabled patients to provide feedback about the acceptability of identifiable data being used without consent, as requested by CAG, prior to the provision of ongoing support. Regarding CAG approval, patients generally understood that using identifiable data without consent could maximise public benefit by improving future outcomes (see patient quotes in Box 1). They also understood that the rarity of blood cancer meant that data were needed at the population level, to accurately inform clinical practice. CAG was reassured by these positive responses and the strong belief that HMRN was sufficiently important to warrant data-use without consent; and the cohort continues to receive support. This PPI led to a revision of the notification material (e.g., posters, leaflets), with clearer statements about the aims and importance of the study, and details about opting out, although this rarely occurs.

**Figure:**
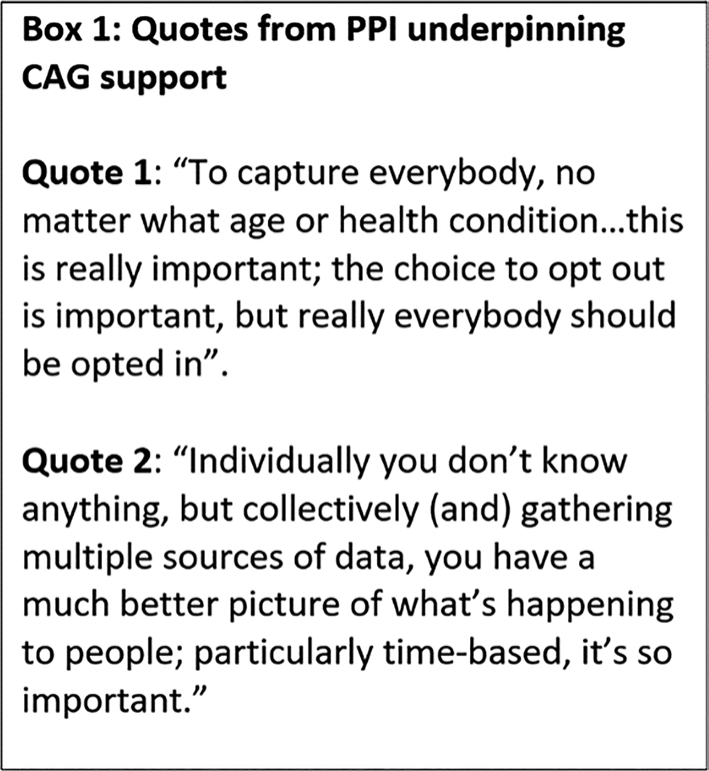

The impact of a recent *Open Day* was clear, with over 200 people attending the event and ~ 90% of respondents reporting ‘excellent’ feedback (see patient quotes in Box 2). Our charity partners shared the event on their websites, and the Support Group (led by CM) managed a dedicated area from which to greet patients and their families. Invitations were distributed via flyers in NHS haematology clinics and in HMRN study packs. The Open Day also benefitted the entire research team, but particularly junior staff who do not usually come into contact with patients and families; although these are the people who are most impacted by their work.

**Figure:**
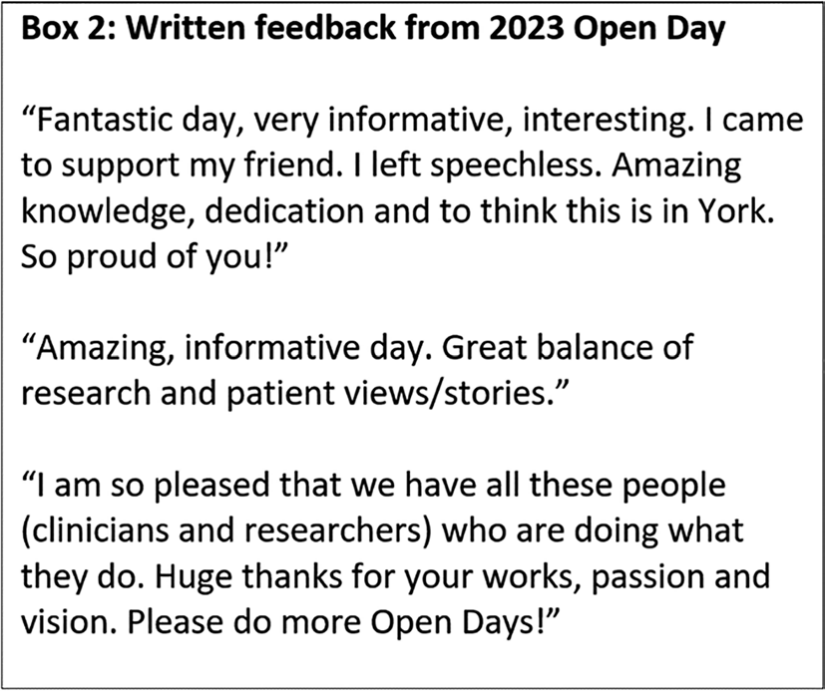

##### Factors for success and challenges

Various factors for success, as well as challenges were identified over the course of developing the cohort’s PPI, as is summarised in Supplementary Material 3. Initially, good PPI was found to require experienced researchers who could identify the right people; enable and maintain good patient-researcher relationships over time; build trust; manage and facilitate meetings and events, and provide timely feedback. Difficult conversations about sensitive issues could arise unexpectedly during PPI sessions, occasionally triggering strong emotions, and good communication and interpersonal skills were found to be essential among facilitators, so they could appropriately manage such circumstances. Signposting has been used in the past when required, for example to haematology Clinical Nurse Specialists in the study area, who are routinely alerted to this possibility.

Establishing the Patient Partnership was crucial to the success of HMRN’s PPI as it provided a mechanism for further contact with patients, for such purposes. It also enabled the selection and inclusion of people with specific blood cancer sub-types in our activities, who could best describe what it was, or is like, to live with their particular type of illness within their own personal, physical and socio-economic contexts. These individuals were able to describe the problems they themselves faced (or still face), and what might improve their lived experiences in the future. However, we have found it difficult to attract people from underserved groups into our PPI, and with this in mind HMRN’s PPI is currently being significantly expanded, with more patient and public members from such groups on our Oversight Committee and sub-committees.

Significant time and effort were required for successful PPI as it took time to build trust and really get to know people who were initially strangers. Consistency was found to be key, and included delivering on goals (as far as possible), and recognising and appreciating the importance of patients as experts with lived experience. HMRN’s expanding PPI has required more resources over time, for example to support dedicated staff who are able to meet the public out of hours, including weekends (e.g., open days, conferences) and evenings (consultations and co-working groups). Money and time were also required for patient (and staff) training, Open Days and other PPI-linked events, and to reimburse expenses and the time dedicated to activities (e.g., meetings preparation). Worsening health status could be challenging for patients, but also impacted researchers, who inevitably developed close relationships with patients and families, particularly if regular contact was maintained, sometimes over many years. This could be heightened if irrevocable deterioration frequently occurred.

In practical terms, the research team found that three facilitators were required for successful face-to-face consultations (e.g., focus group meetings) involving 6–12 people. This ensured sufficient people were available to welcome, direct and chaperone people from the car park, and serve refreshments, particularly if assistance was required, or attendees were nervous or new to such activities, which was often the case. Facilitators were also needed to record the session (written or audio), leave the room and support anyone who became distressed, and manage and steer conversations where necessary to ensure everyone had the opportunity to speak. Parking was always organised and paid for ahead of the event to avoid this becoming stressful, and feedback forms were distributed at the end of each meeting, with responses complied and discussed by the team, and learning points agreed upon and used to improve future activities.

Smaller group meetings were found to be beneficial, and were said to be more comfortable by some people than large events; they could also facilitate relationship building and in-depth discussion. More recently, online focus groups were found to be successful, particularly with smaller groups, and the option of attending an online or face-to-face meeting is now routinely offered, with day-time and evening options to promote inclusivity (see Sect. “Examples of Patient and Public Involvement Across the Research Process”).

## Discussion

This paper provides a case study of the processes undertaken to introduce and establish meaningful PPI within a UK cohort of blood cancer patients. Implemented across two decades, various activities were planned and delivered; many of which are likely to be transferable to other cancers and conditions. In common with other long-term studies, our PPI began with something akin to ‘trial and error’^47^, but has continued to develop and expand, within a discipline that has become essential, innovative and fast-moving. This has occurred alongside growing experience within the team, the gradual increase in patient and public partners (many of who have become long-term collaborators), and a rapid expansion in the team’s access to learning resources, which are often now available online.

Given that 4.7 million people were unpaid carers in England in 2024, many of who were elderly and struggled with their own health [48], benefits were gained from incorporating the lived experiences and perspectives of caregivers (typically kin) in our PPI. This enabled the research team to better understand their role in supporting others; the impact caring has on them; and their own needs, which are often little known and unmet [49–51]. This group is also important because caregivers have extensive knowledge of healthcare services and what could improve their design and delivery [49]. Again, the Patient Partnership enabled us to identify carers for this role.

Generally, people were pleased to take part in the cohort’s PPI activities and willing to help, with some seeing this as their duty and a means of improving future care; although this could be emotional, as well as cathartic, as has been noted by others [52, 53]. The growing PPI literature concurs, reporting that PPI can lead to patients feeling listened to, empowered, valued, gaining in confidence and life skills, doing something meaningful and receiving mutual support from other contributors; as well as becoming more aware and knowledgeable about their condition [53]. Some reported challenges, however, such as overburdening, and a lack of preparation and training; as well as feeling they were not being listened to, or that their views were marginalised [53]; although this was not our experience within HMRN.

Anecdotally, PPI has benefited the cohort’s research, yet in practice some aspects were easier to evidence than others. For example, survey feedback after Open Days was useful; while it was more difficult to quantify less visible benefits (e.g., friendships built over time; people going on to share their skills with other groups). Questions persist about how best to measure the effectiveness and impact of PPI, and even if this is feasible and appropriate [54]. For example, assessment may be constrained by the lack of well-recorded empirical data and metrics [55, 56]; and is rarely tested experimentally [5]. Experiential knowledge (‘knowledge in context’ gained from working directly with patients and the public) has been suggested as a more useful concept [56]. As PPI consumes monetary and non-monetary resources, however, evaluation is needed to provide information about costs, benefits and risks; and the impact and effectiveness of PPI on research processes [5, 55, 57]. A range of tools are now available for this purpose [58], which we will consider using in the future.

Our PPI processes align with those reported in a review of ‘patients as partners’ in health research, which notes the inclusion of patients as members of the research team, supported via meetings, and resulting in improved study design [59]. Although PPI is sometimes criticized for ‘tokenism’ and ‘exclusivity’, and for being inattentive to empowerment, equality, diversity and marginalised or seldom heard groups, the HMRN team has sought to avoid such accusations [60]. Efforts have always been made to conduct the cohort’s PPI in accordance with best practice guidance, in the belief that working in real partnership with people affected by blood cancer will improve our research. PPI remains challenging and time-consuming, however, and the need for resources (time, training, staff and money), should not be underestimated if sufficient capacity is to be built to effectively manage PPI [47, 53, 57].

## Conclusions

The ongoing, ever-expanding and improving PPI associated with the HMRN cohort has enabled patients to collaborate with the research team on many occasions, over two decades. Whether for altruistic or other purposes, PPI has ensured our research is firmly embedded in the lived experiences and real-world circumstances, of people affected by blood cancer. This has resulted in improved research questions and designs, that are better able to involve and engage patients and the public, and underpin meaningful changes that could improve care and achieve better outcomes. The time, effort and resources required to establish and develop such activities should not be underestimated; and it can be difficult to measure the impact of PPI. Finally, it is important to keep abreast of ongoing advances in this rapidly evolving field, and to modify and update practices accordingly.

## Electronic supplementary material

Below is the link to the electronic supplementary material.

Supplementary material 1

## Data Availability

No datasets were generated or analysed during the current study.
