# Supplementary material for: Patient and Public Involvement and Engagement within a UK blood cancer cohort: a case study
Source: Res Involv Engagem. 2026 Jan 20;12:10. doi: 10.1186/s40900-025-00829-6 (PMC12821308; doi:10.1186/s40900-025-00829-6)
Supplement: Supplementary file 1 — Supplementary material 1 [file 40900_2025_829_MOESM1_ESM.docx]

**Supplementary Material 1 - Research Prioritisation Areas**

| **Prioritisation Area** | **Details** |
| --- | --- |
| Treatment related | - Cures! - Non-toxic treatment - Immunotherapy - CART cell therapy advances - Minimising side effects of treatment |
| Mental health, wellbeing and support | - Living with uncertainty - Isolation in leukaemia and stem cell transplant treatment - Quality of life - How other people can help (e.g., family) - How to cope with setbacks |
| COVID related | - Impact of COVID on psychological issues mid/long term, particularly being diagnosed during that time - The effects of COVID on early diagnosis – how advanced is the diagnosis pre and post COVID? - The implications of COVID vaccine on blood cancer patients |
| Organisational | - Hospital/community ‘patient passport’ to improve communication between professionals. This could lead to a much faster treatment of other symptoms following diagnosis. - Joining up all governmental, clinical and industrial outlook of research. Too much red tape for viable/actionable research. |

**Supplementary Material 2 - Examples of verbatim feedback on the HMRN website (via PPI)**

| **Issue** | **Feedback from patients** |
| --- | --- |
| Format | - Font too small, blocks of text too big, fewer words needed - Better colours and navigation needed - Site not welcoming - feels to be aimed at academics - Signposting needed from top level to direct patients to relevant areas - Needs simple layouts and easy-to-find information - Videos/animations/interviews would be useful |
| Content | - Too much information on the ‘landing’ page/each page - Remove some of the numbers and statistics, or carefully choose a few to show - A bit of well-placed humour would be very good - Links to support organisations (e.g., Macmillan) and reputable websites with information about disease, symptoms, treatment, side-effects, hair loss etc. - Links to local support groups - Feedback on current research - Breadth of diseases and symptoms - Patient expectations versus what will or won’t happen to them - How to integrate their disease into their lives (e.g., cancer *can* be chronic) - Accurate, well-sourced information especially on ‘Watch & Wait’, difficulties with treatment decisions, and the meaning of terms such as tiredness in this context |
| Terminology | - Don’t use the term ‘malignancy’ (very important) – use ‘*cancer’* instead |

**Supplementary Table 3 Factors for Success and Challenges of PPI**

| **Factors for success** |
| --- |
| - Establish a group from which to source people for PPI (e.g., the Patient Partnership) |
| - Invite patients with the condition(s) of interest, in accordance with the research question |
| - Consider which stakeholder groups to invite (e.g., patients and caregivers) |
| - Protect the identity of patients and the public |
| - Recognise and appreciate stakeholders as experts with lived experience |
| - Remove barriers that may prevent people from engaging (e.g., offer various meetings/formats) |
| - Reimburse appropriately for time, according to guidance |
| - Provide feedback on all input and activities, so people are aware of the impact of their input |
| - Establish transparent, robust governance practices that include all stakeholders |
| - Experienced facilitators with good communication and interpersonal skills |
| - Ensure that there are sufficient facilitators for the meeting format and content |
| - Acknowledge stakeholder input in publications and reports |
| - Consistently doing what you say you will |
| **Challenges** |
| - Sufficient resources (money and time) |
| - Worsening health status of participants |
| - Facilitators who are able to work evenings and/or weekends, as required |
| - Parking |
